# A Retrospective Review of Secondary Hemophagocytic Lymphohistiocytosis (HLH) and Dengue-associated HLH from a Teaching Hospital in Singapore.

**DOI:** 10.46989/001c.94954

**Published:** 2024-03-18

**Authors:** Allison C. Y. Tso, Sanchalika Acharyya, Sing Zern Fong, Lian K. Lee, Sampath V. Sreekanth, Bingwen E. Fan, Seok W. S. Chan, Kiat H. Ong

**Affiliations:** 1 Haematology Tan Tock Seng Hospital; 2 Clinical Research & Innovation Office Tan Tock Seng Hospital; 3 Laboratory Medicine National University Hospital

**Keywords:** hemophagocytic lymphohistiocytosis, lymphoma, viral infections, dengue

## Abstract

Real-world data on the outcome of Asian patients with secondary hemophagocytic lymphohistiocytosis (HLH), especially on dengue-associated HLH, are limited to small case series. This is a retrospective records review of adult patients with secondary HLH between 2015 and 2020. Thirty-two adult patients were followed up for a median of 6.6 months (range 0.1 – 75 months). 15 had underlying lymphomas, and 12 had viral infections. Hemophagocytosis was seen in 28 of 29 patients with a bone marrow biopsy. 100% and 76.5% of patients with and without an underlying malignancy required HLH-directed therapy and blood product transfusion. 12 of 15 patients with lymphomas were treated with additional chemotherapy. Patients with malignancy-associated HLH had poorer survival than non-malignancy-associated HLH (median overall survival (OS) 1.5 months versus not reached, p-value 0.003). The 1-year survival rates of patients with malignancy-associated HLH, HLH with unknown etiologies, and infection-associated HLH were 0.133 (95% CI: 0.036 – 0.484), 0.400 (95% CI: 0.137 – 1.000) and 0.833 (95% CI: 0.647 – 1.000), respectively. Malignancy significantly increased the risk of death compared to infection-associated HLH (HR 9.37, p-value 0.003). Eight patients were diagnosed with dengue-associated HLH with a median HSCORE of 240 (98-99% probability of HLH). Their mean ferritin was 34,740 ng/mL. Three patients required blood product transfusion, 5 required corticosteroids and/or etoposide, with a median duration of treatment of 31 days. Their overall survival rate was 87.5%. Our study highlights the stark contrast in the survival of secondary HLH patients with and without an underlying malignancy. We also present one of the world’s most extensive case series of dengue-associated HLH.

## Introduction

Hemophagocytic lymphohistiocytosis (HLH) is an aggressive, life-threatening syndrome of excessive cytokine release from CD8+ T cells and macrophage activation, abnormal feedback regulation by NK and cytotoxic T cells, resulting in uncontrolled immune hyperactivation, infiltration of inflammatory cells leading to rapid end-organ failure and death.^1^ Malignancies, infections, or autoimmune conditions can trigger secondary HLH. Recent reviews suggested that the diverse phenotypes arising from various gene mutations lead to the heterogeneity of clinical presentations when triggered by environmental factors.^2^

Clinical features of HLH include fever, hepatosplenomegaly, cytopenias, liver dysfunction, hyperferritinemia, hypofibrinogenemia, hypertriglyceridemia, elevated inflammatory markers and hemophagocytosis. Neurological, pulmonary, and renal abnormalities, hypotension and hyponatremia are not uncommon.^3^ The overall mortality of secondary HLH was reported to be as high as 50-75%.^4^ HLH due to malignancies have an even worse prognosis than those with a non-malignant cause (median overall survival (OS) 1.13 versus 46.53 months, respectively; P <0.0001).^5^ Ferreri et al.^6^ suggested an ethnic predisposition of Japanese and East Asians for malignancy-associated HLH.

Asia-Pacific countries bear three-quarters of the estimated 390 million global dengue cases per year,^7^ and there has been a 600% increase in dengue cases from 1999 to 2019.^8^ The clinical course, optimal treatment, response rate, and outcomes are not well characterized and are usually limited to case reports or small case series. In a paper by Chang et al.,^9^ persistent fever beyond day 7, or a re-emergence of high-grade fever after initial defervescence, prompted investigations for secondary HLH. Patients with dengue-associated HLH exhibit a range of clinical signs and severity. Whilst some patients require HLH-directed therapy, some may not.^10^ The response to dexamethasone at 10mg/m^2^ as per the HLH-94 protocol appeared rapid and could be discontinued as early as 4 to 8 days after initiation,^9^ whilst dexamethasone was tapered after 2 weeks of treatment in other reports.^11^ Chung et al.^11^ conducted a literature search and noted that amongst the 16 cases of dengue-associated HLH, 15 required corticosteroids, 7 required additional intravenous immunoglobulins (IVIG), and the overall survival rate was 75%. A systematic review and meta-analysis on dengue-associated HLH conducted by Giang et al.^12^ found a case fatality rate of 14.6%. Kan et al.^13^ conducted a retrospective study on 180 patients admitted to the intensive care unit with severe dengue; 21 of them were diagnosed with secondary HLH defined by a ≥ 70% probability of HLH as per HSCORE, with a 43% mortality rate (9 of 21 patients).

## Methods

### a. Study design, setting and patients

This is a retrospective medical records review of adult patients aged 21 and above (age of majority in Singapore) diagnosed with secondary HLH between March 2015 and June 2020 at Tan Tock Seng Hospital, Singapore. All cases of suspected HLH in our hospital are referred to the hematology service, where the diagnosis is either confirmed or refuted. This study describes these patients’ demographics, etiologies, therapeutic management, and outcomes. The data cut-off for analysis was on 14^th^ September 2021. Ethics approval and waiver of consent were obtained (DSRB 2020/00833).

### b. Diagnosis of secondary HLH

The diagnosis of secondary HLH was made based on a combination of clinical suspicious laboratory findings and supported by tissue biopsy and the probability of HLH estimated using three validated scoring systems: HLH-2004 trial criteria,^14^ the HSCORE^15,16^; and the 2019 Modified HLH score.^17,18^ HLH-2004 requires fulfillment of at least five of the eight criteria. However, two of the laboratory investigations (low or absent natural killer cell activity and elevated soluble CD25) are not available locally; hence, the maximum possible score is 6 points^19^ (based on fever ≥ 38.5^0^C, splenomegaly, peripheral blood cytopenias, hypertriglyceridemia and/or hypofibrinogenemia, hemophagocytosis in the tissue biopsy, ferritin > 500 ng/mL). Patients scoring 3 or 4 points may still have a high probability of HLH in the appropriate clinical context. According to Fardet et al.,^15^ 169 as a cutoff value for HSCORE corresponded to a sensitivity of 93%, a specificity of 86%, and an accurate classification of 90% of the patients. However, an HSCORE of 169 points corresponds to only a 40 - 54% probability of HLH. To further minimize bias that patients who did not truly have secondary HLH were included in the study, and to increase the credibility of our data, we excluded 2 further patients whose HSCORE was ≤ 170 and who did not undergo bone marrow biopsy or have histological confirmation of hemophagocytosis from the analysis, in line with Kan et al.^13^ where HLH was defined when the HSCORE corresponded to 70% or more probability for HLH.

### c. Data collection

The data we collected include patient demographics and laboratory parameters, including complete blood count (FBC), fibrinogen, lactate dehydrogenase (LDH), liver function (aspartate aminotransferase (AST) and alanine transaminase (ALT)), triglyceride and ferritin). We recorded the presence of fever, hepatosplenomegaly, hemophagocytosis in the bone marrow biopsies, the score for the three validated HLH scoring systems, indications and duration of treatment, time to resolution of clinical and laboratory parameters, the need for additional therapy (including blood product transfusion, chemotherapy, or stem cell transplantation), and clinical outcomes including the cause of death and the overall survival (OS).

### d. Statistical analysis

The distribution of baseline demographic characteristics, clinical presentations, treatment, and outcomes were summarized using appropriate statistical measures and further compared between malignancy- and non-malignancy-associated HLH. OS was defined as the time from HLH diagnosis to death from any cause or date of last follow-up. Survival probabilities over time were estimated using the Kaplan–Meier method, and their distributions between malignancy and non-malignancy-associated HLH were compared using a log-rank test. Hazard ratios (HR) and 95% confidence intervals (CI) were estimated using Cox proportional hazard models. A similar analysis was conducted to estimate and compare the OS between malignancy, infection-associated HLH, and HLH of unknown etiology. In all tests, p < 0.05 was considered significant. All statistical analyses were performed in R version 4.1.1; the package “survival” was used for survival analysis. A subgroup analysis was conducted for dengue-associated HLH cases.

## Results

### Demographics and patient characteristics

32 patients (17 females, 15 males) aged 23 to 80 years (mean 53.4 years; standard deviation 18.8 years) were identified. The majority were Chinese (71.9%), followed by Malay (15.6%), Indian (6.3%), and other (6.3%) ethnicities, mirroring Singapore’s ethnic demographics. The distributions of these characteristics were comparable between malignant and non-malignant HLH groups (table 1). Of the 32 patients, 15 (46.9%) had underlying lymphomas (aggressive B cell (n=4), aggressive T cell (n=5), aggressive NK/T cell (n=4), aggressive lymphoma (not otherwise specified) (n=1), low-grade (marginal zone) in possible transformation to aggressive lymphoma (n=1)). Twelve patients (37.5%) had underlying viral infections (8 dengue, 2 Epstein-Barr virus (EBV), 1 parvovirus, 1 unknown virus (a patient with self-limiting viral symptoms which preceded the diagnosis of HLH; however, no virus was isolated. She remained well (OS 75 months) with no relapses) (table 2). No definite underlying cause was identified in 5 patients. Drug-induced HLH was suspected in two of the 5 patients (one was admitted with opioid overdose and was postulated as a trigger in the second patient). These 5 patients were included as secondary HLH, as their median age was 52 (making primary HLH less likely), they had no known history or family history of HLH, no malignancy or autoimmune disease were identified during their workup, and all had features of hemophagocytosis without lymphoma or other malignancies in their bone marrow biopsies. Interestingly, autoimmune trigger as a cause was not identified in our patient cohort. Twenty-nine patients (90.6%) had a bone marrow biopsy performed.

**Table 1.** Distribution and comparison of demographic and clinical characteristics for the study population

Characteristics	**Underlying Malignant cause (N=15)**	**Underlying Non-Malignant cause (N=17)**	P value^*^
Sex			
Male	9 (60%)	6 (35.3%)	0.162
Female	6 (40%)	11 (64.7%)	
Age			
Median (IQR)	65 (30.5)	44 (30)	0.142
Mean (SD)	59.3 (17.7)	48.3 (18.8)	0.101
Race			
Chinese	14 (93.3%)	9 (52.9%)	0.078
Malay	1 (6.7%)	4 (23.5%)	
Indian	0	2 (11.8%)	
Eurasian	0	1 (5.9%)	
Filipino	0	1 (5.9%)	
HLH Scores			
HLH-2004 6 points	4 (26.7%)	3 (17.6%)	
HLH-2004 ≥5 points	11 (73.3%)	9 (52.9%)	0.163
HLH-2004 4 points	0	4 (23.5%)	
HLH-2004 3 points	0	0	
HLH-2004 2 points	0	1 (5.9%)	
Diagnostic by Modified HLH	14 (93.3%)	16 (94.1%)	0.927
Median HSCORE	245 (>99% probability HLH)	235 (98-99% probability HLH)	0.202
Treatment			
First line HLH-directed treatment			
None	0	4 (23.5%)	0.102
Corticosteroids only	5 (33.3%)	7 (41.2%)	
Dexamethasone + Etoposide	10 (66.7%)	6 (35.3%)	
Median duration of treatment (IQR), in days	23 (17)	15 (31)	0.786
Reasons for stopping treatment			
Responded to therapy	2 (13.3%)	8 (47.1%)	0.017
Complications/side effects of therapy	1 (6.7%)	0	
Switched to chemotherapy	3 (20%)	0	
Death	9 (60%)	5 (29.4%)	
Data not available	0	4 (23.5%)	
Requiring additional Second Line (Anakinra and IVIG)	1 (6.7%)	1 (5.9%)	
Requiring systemic Chemotherapy	12 (80%)	0	
Requiring Bone Marrow Transplant	2 (13.3%)	0	
Required Blood Product Transfusion	15 (100%)	12 (70.6%)	0.022
Outcomes			
Admission to High Dependency/Intensive Care	8 (53.3%)	7 (41.2%)	0.492
Mortality	14 (93.3%)	5 (29.4%)	<0.001

*p values are obtained from two independent sample t tests or Mann-Whitney U tests for continuous variables and chi-squared or Fisher exact test for categorical variables.

**Table 2.** Distribution of underlying subtypes of HLH and their outcomes.

**Underlying Cause**	**Total N = 32**	**Admission to High Dependency/Intensive Care**	**Inpatient mortality**
**Malignancy**	**15 (46.8%)**		
Aggressive B cell lymphoma	4 (26.7%)	2 (50.0%)	4 (100%)
Aggressive T cell lymphoma	5 (33.3%)	3 (60.0%)	4 (80.0%)
Aggressive NK/T cell lymphoma	4 (26.7%)	1 (25.0%)	4 (100%)
Aggressive lymphoma, unknown subtype (NOS)	1 (6.7%)	1 (100%)	1 (100%)
Non-aggressive lymphoma	1 (6.7%)	1 (100%)	1 (100%)
**Underlying Viral Infection**	**12 (37.5%)**		
Dengue	8 (66.7%)	1 (12.5%)	1 (12.5%)
Epstein Barr Virus (EBV)	2 (16.7%)	2 (66.7%)	2 (66.7%)
Parvovirus B-19	1 (8.3%)	0	0
Unknown virus	1 (8.3%)	0	0
**No identifiable underlying cause**	**5 (15.6%)**	5 (100%)	3 (60%)

All patients had a fever ≥ 38.5^0^C (range 38.5 to 40.8^0^C). Twelve of 32 patients (37.5%) had splenomegaly. 29 (90.6%) had at least two lines of cytopenias characterized by Hb < 9 g/dL, platelet < 100 x 10^9^/L, and neutrophil < 1 x 10^9^/L. The median values of hemoglobin (Hb), platelet, and neutrophil counts were 7.0 g/dL, 24 x 10^9^/L, and 1.13 x 10^9^/L, respectively. Nineteen (59.4%) patients had elevated fasting triglyceride (defined as > 2.99 mmol/L) with a median of 3.90 mmol/L, 71.9% had hypofibrinogenemia (defined as < 1.5 g/L) with a median of 1.05 g/L, and 96.9% had transaminitis (defined as above the laboratory reference range) with a median AST of 612 U/L (range 54 – 11,614 U/L) and ALT of 351 U/L (range 51 – 7,361 U/L). All patients had elevated LDH (median 2,013 U/L). Quantified ferritin ranged from 1,500 - 113,500 ng/mL (mean 30,828 ng/mL). Of the 29 patients with a bone marrow biopsy, 28 (96.6%) demonstrated hemophagocytosis. Our patient cohort’s median “HLH-2004” score was 5 (of a maximum of 6 points). 30 (93.8%) patients had HLH according to the modified HLH criteria. The average HSCORE amongst our cohort was 245.1, indicating a > 99% probability for HLH.

### Treatment

**Figure 1.**
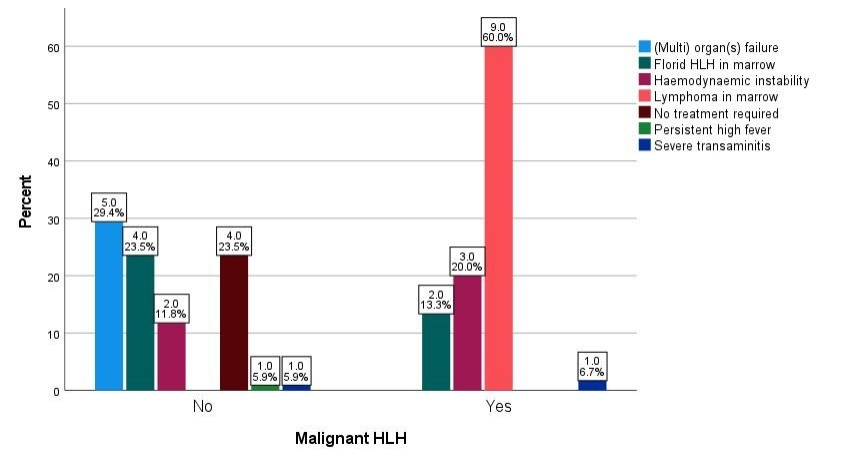
Distribution of indications for starting HLH treatment, apart from HLH in marrow and HLH parameters

The respective primary hematologist decided to initiate HLH-directed therapy. Apart from the patient’s clinical status, abnormal laboratory parameters, and/or hemophagocytosis in the bone marrow biopsies, key additional reasons for the treating physician to initiate HLH-directed therapy are summarized in **Figure 1**. The presence of lymphoma in the bone marrow was the leading indication (for 60% of patients) within the malignant group, followed by hemodynamic instability (20%) and florid hemophagocytosis in the bone marrow (13.3%). For the non-malignant group, the leading indication was organ or multiorgan failure (29.4%), followed by florid hemophagocytosis in the bone marrow (23.5%) and hemodynamic instability (11.8%).

HLH-directed therapy was administered to 28 patients (87.5%), including all (n=15) cases of malignancy-associated HLH, all (n=5) cases with unknown etiology, and 8 of 12 cases of infection-associated HLH. 12 of the 28 patients were treated with dexamethasone or other corticosteroids only. 16 were treated with a combination of dexamethasone and etoposide chemotherapy, and 2 of these required second-line HLH-directed therapy [anakinra and Intravenous immunoglobulin (IVIG)], as they did not respond to first-line therapy, with worsening clinical status. Four patients did not require any treatment with spontaneous resolution of HLH; they all had infection-associated HLH comprising 3 cases with dengue and 1 with EBV infection.

Among 17 patients without an identified underlying malignancy, 76.5% required HLH-directed therapy and blood product transfusion (in contrast to 100% of patients with an underlying malignancy). The median duration of treatment for the non-malignant and malignant groups was 15 and 23 days, respectively. 15 patients required high dependency (HD) or intensive care (ICU) admission. This comprised 8 patients with an underlying malignancy, 2 with underlying infection, and 5 with unknown underlying etiology.

### Resolution of symptoms and clinical / laboratory parameters

Resolution of fever (defined as a temperature of < 38^0^C) occurred within one day for a quarter of patients; for others, the median time taken for the fever to resolve was 5 days (minimum 2, maximum 20 days). Laboratory parameters such as low hemoglobin (Hb), low platelet, and high ferritin levels were not resolved for nearly half of the patients at the time of analysis. Some patients did not undergo repeat blood tests as out-patients to confirm the resolution of previously abnormal laboratory parameters. For example, amongst the 10 patients with infection-associated HLH who are alive, 5 did not have repeat ferritin to document normalization, as the parameter was either already on a downward trend or near normalization. The distribution of time taken for the resolution of symptoms or laboratory parameters by the malignant and non-malignant groups is presented in Table 3. We observed a general trend for longer duration of symptoms in patients with malignancy-associated HLH, except for raised AST and ALT. Similarly, there was generally a higher proportion of patients with unresolved symptoms in the malignant group, except for hypofibrinogenemia. We did not compare these distributions statistically, considering multiple testing time points.

**Table 3.** Distribution and comparison of presenting symptoms, blood parameters and their time to resolution

**Characteristics**	**Underlying Malignant cause (N=15)**	**Underlying Non-Malignant cause (N=17)**	P value^*^
**Temperature in ℃, mean (SD)**	39.5 (0.7)	39.7 (0.9)	0.462
Days to lysis of fever (= <38℃)			
1 day	4 (26.7%)	4 (23.5%)	0.623
≥ 2 days	8 (53.3%)	7 (41.2%)	
Median time to lysis (min - max)	6 (2 - 20)	4 (2 - 8)	
Data not available	3 (20.0%)	6 (35.3%)	
**Ferritin in ng/mL, median (min - max)**	17764 (3405 - 113500)	19459 (1500 - 78425)	0.655
Days taken to lower ferritin to < 500			
Median (min - max), n	23, n = 1	51 (16 - 103), n = 7	
Not resolved, n (%)	11 (73.3%)	5 (29.4%)	
Data not available, n (%)	3 (20%)	5 (29.4%)	
**Hb in g/dL, mean (SD)**	6.57 (0.83)	8.67 (3.05)	0.013
Hb ≤ 9 g/dL, n (%)	15 (100%)	12 (70.6%)	0.022
Days taken for Hb to recover to > 9 g/dL			
Median (min - max), n	19 (6 - 30), n = 4	14 (8 - 69), n = 5	
Not resolved, n (%)	9 (60%)	5 (41.7%)	
Data not available, n (%)	2 (13.3%)	2 (16.7%)	
**Platelet ×10^9^/L, median (min - max)**	16 (4 - 188)	49 (8 - 197)	0.132
Patients with thrombocytopenia	n=14	n=16	
Days taken for platelet to recover to >110			
Median (min - max), n	16 (5 - 27), n = 4	4 (1 - 14), n = 11	
Not resolved, n (%)	9 (64.3%)	5 (31.3%)	
Data not available, n (%)	1 (7.1%)	0	
**Days taken for resolution of WBC to normal laboratory reference range**			
Median (min - max), n	16 (8 - 42), n = 5	6 (1 - 18), n = 12	
Not resolved, n (%)	8 (53.3%)	2 (11.8%)	
Within normal range, n (%)	2 (13.3%)	3 (17.6%)	
**Fibrinogen in g/L, median (min - max)**	0.9 (0.5 – 4.7)	1.2 (0.4 – 4.0)	0.153
Fib <1.5 g/L, n (%)	13 (86.7%)	10 (58.8%)	0.087
Days taken for Fibrinogen to normalise			
Median (min - max), n	15 (1 - 30), n = 9	9 (1 - 27), n = 7	
Not resolved, n (%)	3 (23.1%)	3 (30.0%)	
Data not available, n (%)	1 (7.7%)	0	
**AST (U/L), median (min - max)**	533 (47 - 8081)	975 (89 - 11614)	0.202
**ALT (U/L), median (min - max)**	232 (49 – 1183)	545 (129 - 7361)	0.044
**Hepatitis (raised AST/ ALT), n (%)**	14 (93.3%)	17 (100%)	0.469
Days taken for resolution of AST/ALT			
Median (min - max), n	11.5 (7 - 37), n = 8	21 (10 - 55), n = 11	
Not resolved, n (%)	7 (46.7%)	4 (23.5%)	
Data not available, n (%)	0	2 (11.8%)	

### Response to HLH-directed therapy

The response to first-line HLH-directed therapy amongst the malignant group was generally poor and mostly non-sustained (table 1). Only 2 (13.3%) of the 15 patients responded. For one of the responders, treated for 59 days with dexamethasone, relapse occurred 2 months later, with a recurrence of fever and abnormal laboratory parameters. She was subsequently diagnosed with aggressive T-cell lymphoma and underwent chemotherapy but passed away from complications of stem cell transplantation. The second responder (with low-grade lymphoma in the possible transformation to high-grade lymphoma) responded to dexamethasone and etoposide, remained on treatment for 33 days, and was discharged from the hospital. 6 months later, his HLH recurred when he was diagnosed with aggressive lymphoma, which became refractory to chemotherapy, and death ensued shortly thereafter. Nine of the 15 patients died shortly after initiating HLH-directed therapy (median duration on treatment was 20 days, range 5 - 45 days), 2 from HLH, and 7 from lymphoma progression. One further patient who was treated with dexamethasone, etoposide and chemotherapy for lymphoma was transferred to another hospital. He was reported to have suffered from treatment complications, and the response to HLH-directed therapy cannot be ascertained. Three patients discontinued HLH-directed therapy after a median treatment duration of 16 days (range 10 – 28 days) and were switched to chemotherapy. Amongst these 3 patients, one died of sepsis whilst on chemotherapy, and one was subsequently transferred to another hospital for further treatment, but passed away (cause unknown) 4.8 months after the initial admission with HLH. The third underwent a subsequent stem cell transplant and was alive on the day of the last follow-up (OS 18.31 months). Amongst the 15 patients with underlying lymphoma, 12 (85.7%) were also treated with systemic chemotherapy. Two patients with aggressive T-cell lymphoma underwent stem cell transplantation after systemic chemotherapy (table 1).

The response to first-line HLH-directed therapy amongst the non-malignant group was more favorable. 13 of the 17 patients received first-line HLH-directed therapy (dexamethasone +/- etoposide). Eight (61.5%) of the 13 responded (median duration on treatment was 40 days) and are all alive (median OS 35.7 months). Five patients died (median duration on treatment was 11 days) with a median OS of 0.5 months. Amongst the 12 patients with infection-associated HLH, 8 received treatment (median duration on treatment was 23 days) and 6 patients (75%) responded, whilst 2 died from HLH and pneumonia, respectively.

Two patients (one from each the malignant and non-malignant group) did not respond to first-line HLH-directed therapy and were treated with second-line HLH-directed treatment - anakinra and IVIG. Neither responded, and both died from pneumonia and HLH, respectively, within a month of their admission.

### Outcomes

The median follow-up was 6.6 months (range 0.1 – 75 months). Nineteen patients (59.4%) were deceased, including 14 and 5 patients with and without an underlying malignancy, respectively (table 1).

**Figure 2.**
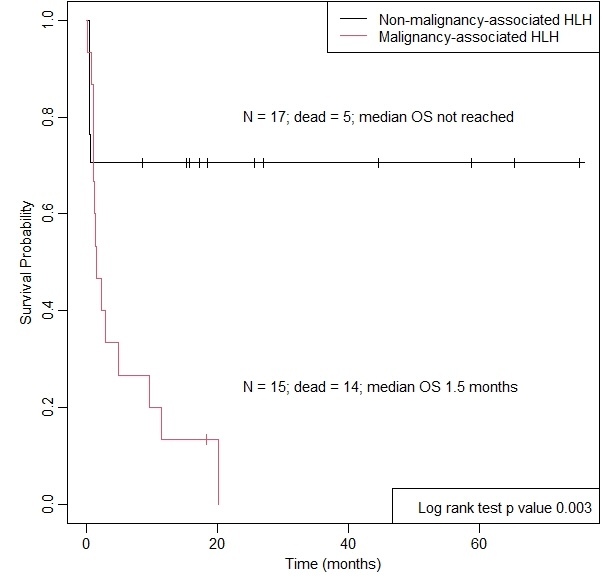
Kaplan–Meier curve for overall survival of patients with malignancy versus non-malignancy associated hemophagocytic lymphohistiocytosis

**Figure 3.**
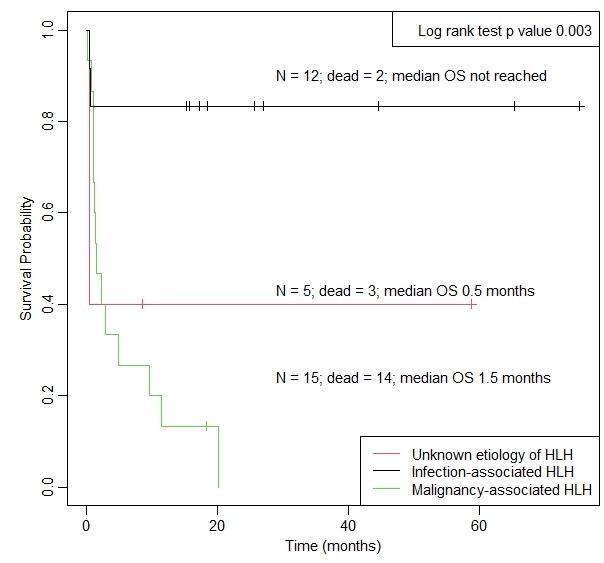
Kaplan–Meier curve for overall survival of patients with hemophagocytic lymphohistiocytosis; a comparison of three subtypes

Malignancy-associated HLH patients had poorer survival than non-malignancy-associated HLH (median OS 1.5 months versus median OS not reached, p=0.003; figure 2). When we distinguish non-malignancy-associated HLH into two further etiologies, infection-associated HLH had better survival outcomes than malignancy-associated HLH and HLH of unknown etiologies (figure 3). The 1-year survival rates of patients with malignancy-associated HLH, HLH with unknown etiologies, and infection-associated HLH were 0.133 (95% CI: 0.036 – 0.484), 0.400 (95% CI: 0.137 – 1.000) and 0.833 (95% CI: 0.647 – 1.000), respectively.

Malignancy significantly increased the risk of death when compared to infection-associated HLH (HR 9.37, 95% CI: 2.1 – 42.5, p=0.003). There was no evidence of a significant difference in the hazard rate between malignant-associated HLH and HLH of unknown etiology (HR 1.22, 95% CI: 0.34 – 4.34, p=0.753).

**Figure 4.**
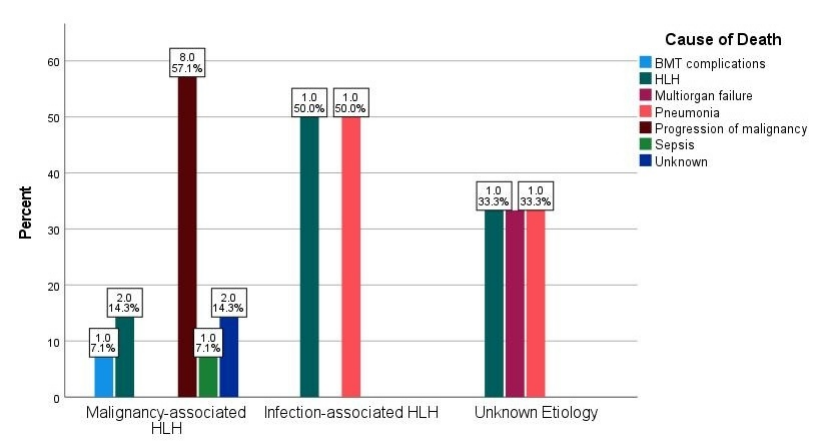
Distribution of cause of death by three underlying etiologies of HLH

The causes of death in the three etiological groups are presented in Figure 4. Disease progression was the major cause for the malignant group (57.1%), while one patient (7.1%) died from bone marrow transplant complications and sepsis. Among the two deaths in the infection-associated HLH group, one patient died from pneumonia and the other from HLH. Three deaths in the unknown etiology group were each caused by HLH, pneumonia, and multiorgan failure, respectively. The cause of death of 2 patients with malignancy-associated HLH was not known.

### Dengue-associated HLH Subgroup Analysis

Eight patients were diagnosed with HLH secondary to dengue fever; three did not undergo bone marrow examination. All 5 patients who underwent bone marrow examination had evidence of hemophagocytosis. These patients scored a median of 4 points according to HLH-2004 criteria. Seven patients satisfied the Modified HLH score. Their median HSCORE was 240, indicating a 98-99% probability of HLH.

Median values of the measured temperature, hemoglobin, platelet and neutrophil counts were 40℃, 11.9 g/dL, 52.5 x 10^9^/L, and 1.02 x 10^9^/L, respectively. Five patients (62.5%) had at least two lines of cytopenias. Splenomegaly and hepatomegaly were present in 3 (37.5%) and 2 (25%) patients, respectively. All patients had elevated LDH (median 3,213 U/L), ferritin (median 23,505 ng/mL, mean 34,740 ng/mL) and hepatitis (median ALT 500 U/L, AST 679 U/L). Five patients (62.5%) had elevated fasting triglyceride (median 4.3 mmol/L), and three patients had hypofibrinogenemia (median 0.9 g/L).

Three patients did not require any HLH-directed therapy with spontaneous resolution of HLH and were discharged after a median admission duration of 10 days. Their median OS was 17.2 months. Three patients required corticosteroids only, and 2 received corticosteroids (dexamethasone) with etoposide chemotherapy. None of the dengue-associated HLH patients required second-line HLH therapy. Only 3 of these 8 patients received blood product transfusion. Besides clinical findings and abnormal laboratory parameters, the most common additional reason for the treating physician to initiate HLH-directed therapy was the presence of florid HLH in the bone marrow (3 of 5 patients). Four (80%) of the 5 patients who received first-line HLH-directed therapy responded to the treatment (median duration of treatment was 31 days). The single patient who required high dependency / intensive care admission succumbed to the disease within a month of diagnosis (table 4). Seven of the 8 patients with dengue-associated HLH are still alive, with a median OS of 18.41 months.

**Table 4.** Descriptive summary of Dengue-associated HLH (n=8).

Sub	Gender	Age	Ethnicity	Tissue Biopsy demonstra-ting HLH	HLH 2004 Score	Modified HLH Criteria (Yes/No)	HSCORE	HSCORE probability for HLH %	First line HLH-directed therapy	Indication for therapy	HD/ ICU	Status	Cause of death	OS in months
1	Male	33	Chinese	Y (BM)	4	Yes	209	88 to 93	Dexamethasone or equiv steroids only	Florid HLH in marrow	No	Alive	Alive NA	25+
2	Female	33	Chinese	Y (BM)	5	Yes	281	>99	Dexamethasone or equiv steroids only	Florid HLH in marrow	No	Alive	Alive NA	26+
3	Female	61	Chinese	Y (BM)	4	Yes	251	>99	None	NA (no treatment required)	No	Alive	Alive NA	18+
4	Female	36	Chinese	Y (BM)	6	Yes	289	>99	Dexamethasone or equiv steroids + Etoposide	severe transaminitis	No	Alive	Alive NA	65+
5	Female	23	Chinese	Unknown	2	No	192	80 to 88	None	NA (no treatment required)	No	Alive	Alive NA	17+
6	Male	37	Malay	Unknown	4	Yes	235	98 to 99	Dexamethasone or equiv steroids only	persistent high fever	No	Alive	Alive NA	15+
7	Female	77	Eurasian	Y (BM)	5	Yes	245	>99	Dexamethasone or equiv steroids + Etoposide	Florid HLH in marrow	Yes	Dead	HLH	1
8	Male	63	Chinese	Unknown	4	Yes	220	93 to 96	None	NA (no treatment required)	No	Alive	Alive NA	15+

BM = bone marrow. OS = overall survival. HD = high dependency unit. ICU = intensive care unit. NA = not applicable

## Discussion

Our study demonstrated the stark contrast in the OS of patients with malignancy and non-malignancy-associated HLH (median OS 1.5 months versus not reached, p=0.003), where 93.3% and 29.4% of patients, respectively, passed away at the time of data analysis. Patients with malignancy-associated HLH are more likely to require etoposide, second-line HLH treatment, transfusion, and high dependency or intensive care admission. Treating the underlying malignancy is paramount for a chance of survival. There may also be a need to repeat the workup in search of an occult malignancy in patients whose HLH recurs after an initial response to HLH-directed therapy. The outcome of malignancy-associated HLH in our study was worse than that of MD Anderson Cancer Centre’s experience,^20^ possibly due to our small number of patients (6.7% and 20% OS rate, respectively). However, the median survival was similar at 1.5 and 1.43 months, respectively).

There are several limitations in our retrospective study. We only included cases of HLH known to our hematology service, and this is dependent on referrals by the general physicians. Hence, the true incidence of secondary HLH in our hospital over the last 5 years is likely much higher. Although diagnosing secondary HLH is generally objective, based on a combination of clinical findings, laboratory parameters, and likelihood of HLH based on the 3 scoring systems, there may be some heterogeneity in the diagnosis, as not all (3 of 32) patients underwent a bone marrow biopsy. We mitigated this by excluding patients with an HSCORE of 170 or below who did not undergo bone marrow biopsy demonstrating the presence of hemophagocytosis. That said, hemophagocytosis is not very specific for HLH as it can be seen in patients with severe sepsis, nor is it a mandatory criterion for diagnosing HLH.^21,22^ As NK cell activity and elevated soluble CD25 tests are unavailable in Singapore, this would affect the scoring by HLH-2004 trial criteria. The HSCOREs of patients with dengue-associated HLH may also be influenced by the degree of thrombocytopenia, a characteristic of dengue infection. By inference, depending on the treating hematologist, there is also likely some degree of heterogeneity in the management of HLH, such as when to initiate treatment, whether to add etoposide to dexamethasone as first-line HLH-directed therapy, and when to initiate second-line HLH therapy. The periodic nature of reviewing patients after their discharge from the hospital meant the earliest date for normalization of abnormal laboratory parameters would not be easily captured. Likewise, some patients did not undergo repeat blood tests to demonstrate complete resolution of their previously deranged laboratory parameters if those parameters were already improving, or near normal. There were also some missing data due to the migration of the patient electronic medical records system. Furthermore, the cause of death of two patients who passed away outside of our hospital was not obtainable. In our cohort, two patients with malignancy-associated HLH opted for palliative care instead of chemotherapy for their known aggressive lymphomas; this would also influence our cohort’s median OS.

Despite the more favorable outcome of patients with HLH attributed to a non-malignant cause, our study, in line with Kan et al.’s,^13^ has demonstrated that dengue-associated HLH can behave very aggressively with adverse outcomes. The median ferritin in our dengue cohort was higher than patients with malignancy-associated HLH (23,505 ng/mL and 17,764 ng/mL, respectively), and most (62.5%) of our patients required HLH-directed therapy. Although patients with dengue-associated HLH^9,10,13^ are usually treated with corticosteroids +/- IVIG +/- anakinra, without etoposide chemotherapy, our experience (with 2 of 5 patients), as well as other reports in the literature,^13,23,24^ suggested that some patients do require etoposide in addition to corticosteroids. On the other hand, some patients with dengue-associated HLH may not require HLH-directed therapy and can be supported with spontaneous resolution of their disease (3 of 8 patients in our cohort). Our 3 cases would add to the paucity of similar experiences in the literature.^25^ The criteria for initiating and the optimal treatment are also not well characterized and will require further studies. Although the number of patients in our retrospective study is small, it is still considerable for a relatively rare condition. Based on the PubMed literature search, our study records the second-largest series of adults with dengue-associated HLH, with one (12.5%) fatality. Co-existing HLH should be considered in patients with severe dengue, especially in countries where dengue is endemic. The incidence of dengue-associated HLH may only be realized with better awareness and recognition.

In conclusion, prompt recognition amongst physicians likely to encounter HLH, early referral to hematologists, prompt initiation of HLH-directed and supportive therapies, and managing the underlying triggers are key to improving the outcome of this aggressive and fatal disease.

### Competing Interests

All authors certify that they have no affiliations with or involvement in any organization or entity with any financial or non-financial interest in the subject matter or materials discussed in this manuscript. No funding was received to conduct this study.

### Ethics approval and consent

This study was approved by the NHG (National Healthcare Group) DSRB (domain-specific review board) independent ethics committee (approval number: 2020/00833). The ethics committee waived informed consent due to the study’s retrospective nature. All procedures were according to the ethical standards in the 1964 Declaration of Helsinki and its later amendments.

### Authors’ contribution

Conceptualization: Allison C. Y. Tso (Lead). Investigation: Allison C. Y. Tso (Lead). Methodology: Allison C. Y. Tso (Lead). Formal Analysis: Allison C. Y. Tso (Lead), Sanchalika Acharyya (Supporting). Writing – original draft: Allison C. Y. Tso (Lead). Software: Sanchalika Acharyya (Lead). Writing – review & editing: Sanchalika Acharyya (Equal), Sing Zern Fong (Equal), Lian K. Lee (Equal), Sampath V. Sreekanth (Equal), Bingwen E. Fan (Equal), Seok W. S. Chan (Equal), Kiat H. Ong (Equal). Data curation: Sing Zern Fong (Equal), Lian K. Lee (Equal), Sampath V. Sreekanth (Equal), Bingwen E. Fan (Equal), Seok W. S. Chan (Equal), Kiat H. Ong (Equal).

### Data Availability Statement

Further data will be available upon request to the corresponding author.
